# Antibacterial and Anti-inflammatory Potential of *Morus alba* Stem Extract

**DOI:** 10.2174/1874210601812010265

**Published:** 2018-03-30

**Authors:** Ichaya Yiemwattana, Niratcha Chaisomboon, Kusuma Jamdee

**Affiliations:** 1 Department of Preventive Dentistry, Faculty of Dentistry, Naresuan University, Phitsanulok, Thailand; 2 Research Laboratory, Faculty of Dentistry, Naresuan University, Phitsanulok, Thailand

**Keywords:** *Morus alba* L., Interleukin (IL)-6 and IL-8, Lipopolysaccharide, PDL fibroblasts, Inflammatory cytokines, Periodontal diseases

## Abstract

**Background:**

Periodontitis, a chronic inflammatory disease, is the leading cause of tooth loss in adults. Evidence for the anti inflammatory activity of *M. alba* Stem Extract (MSE) in periodontal disease is limited.

**Objective:**

The study aimed to investigate the inhibitory effect of MSE on the growth of periodontopathic bacteria and expression of interleukin (IL)-6 and IL-8 in *Porphyromonas gingivalis* Lipopolysaccharide (LPS)-stimulated human Periodontal Ligament (hPDL) fibroblasts.

**Methods:**

The antimicrobial activities of MSE were tested against *P. gingivalis* and *Aggregatibacter actinomycetemcomitans* by the disk diffusion, the minimum inhibitory concentration and the minimal bactericidal concentration methods. Cytotoxicity of *P. gingivalis* LPS and MSE on hPDL fibroblasts was determined by MTS assay. The expression of cytokines (IL-6 and IL-8) mRNA and proteins in hPDL fibroblasts was measured using the reverse transcription-qPCR and enzyme-linked immunosorbent assay, respectively.

**Results:**

MSE exhibited antibacterial activities against *P. gingivalis* and *A. actinomycetemcomitans* with the zones of inhibition of 10.00 ± 0.33 mm and 17.33 ± 0.58 mm, respectively. MIC and MBC values for MSE against *P. gingivalis *were 62.5 μg/ml. The MIC and MBC values against *A. actinomycetemcomitans* were 250 μg/mL and 500 μg/ml, respectively. *P. gingivalis* LPS was shown to mediate the expression of pro-inflammatory cytokines in hPDL fibroblasts. However, treatment with MSE concentrations of 2.5 and 5.0 μg/ml significantly suppressed *P. gingivalis* LPS-induced IL-6 and IL-8 mRNA and protein expression (*p*< 0.05).

**Conclusion:**

The present study demonstrates that MSE has antibacterial activity against two putative periodontal pathogens. MSE suppressed IL-6 and IL-8 expression in *P. gingivalis* LPS-stimulated hPDL fibroblasts, indicating a possible anti-inflammatory effect. Thus, it is a potential adjunctive agent for the treatment of periodontitis.

## 1 INTRODUCTION

Periodontitis is characterized by inflammatory destruction of the supporting tissues of the teeth [1]. The etiology of this disease is related to uncontrolled bacterial pathogen-host interactions, resulting in the alteration of resident cells in the periodontium [2]. A few Gram-negative bacterial species have been specifically associated with periodontitis, including *Porphyromonas gingivalis* and *Aggregatibacter actinomycetemcomitans* [3]. *P. gingivalis* Lipopolysaccharide (LPS) is one of the key virulent attributes, which is significantly involved in periodontal pathogenesis [4, 5]. *P. gingivalis* LPS acts as a potent stimulus to a variety of host cells, which subsequently results in the expression of inflammatory cytokines leading to the development and progression of the related host immune response in periodontal diseases [6].

Human Periodontal Ligament (hPDL) fibroblasts, the primary cultured cell of the ligament, are responsible for the formation, repair and maintenance of the extracellular matrix of the hPDL. These cells are involved in inflammatory and immune processes leading to cytokine (*i.e.*, interleukin (IL)-1 or IL-6) or chemokine (*i.e.*, IL-8) release which could further enhance inflammation [7, 8] and finally leading to periodontal destruction *via* Matrix Metalloproteinases (MMPs) [9]. IL-6 is a proinflammatory mediator that activates host cells and in turn, leads to bone resorption [10]. IL-8 is a chemokine produced by a variety of tissues and blood cells, and it is a potent inducer of neutrophil chemotaxis following activation in inflammatory regions [11]. *P. gingivalis* LPS has been shown to stimulate the production of IL-6 and IL-8 by hPDL fibroblasts [12, 13], suggesting that these mediators are involved in the pathogenesis of periodontitis.

*Morus alba* L., also known as mulberry or Mhon in Thai has been widely cultivated in Thailand, particularly for the leaves to feed silkworms. Its leaves, fruit and bark have long been used in traditional medicine for the treatment of fever, improvement of eyesight, joint strengthening and lowering blood pressure [14]. The amount of oxyresveratrol (trans-2,3`,4,5`-tetrahydroxystilbene), a natural chemical compound found in *M. alba* extract, in various parts of mulberry tree, is different and the ethanolic extract obtained from stems has the highest amount of this bioactive compound oxyresveratrol compared to the extract obtained from twigs, with the least amount in the leaves [15]. It has been reported that oxyresveratrol from *M. alba* possesses anti-oxidative and radical scavenging activities. It has also been reported to have anti-inflammatory activity by inhibiting the production of nitric oxide and prostaglandin E2, Inducible Nitric Oxide Synthase (iNOS) expression, and NF-ĸB activity in the LPS-activated RAW 264.7 macrophage cells [16]. In addition, *M. alba* stem extract can inhibit nitric oxide production *via* suppression of both the iNOS mRNA and protein. It was also found to inhibit the expression of COX-2 mRNA in LPS-induced RAW 264.7 cells [15]. The other components in MSE such as prenylated flavonoids (*i.e.* kuwanon E) may have anti-inflammatory activity through the inhibition of NO production [17]. However, the anti-inflammatory activity of MSE in hPDL fibroblasts and antibacterial activity have not yet been determined. Therefore, this study aimed to investigate the inhibitory effect of MSE on the growth of periodontopathic bacteria and expression of IL-6 and IL-8 in *P. gingivalis* LPS-stimulated hPDL fibroblasts.

## 2 MATERIALS AND METHODS

### 2.1 Plant Materials and Preparation of Plant Extract

*M. alba* stems were obtained from the Queen Sirikit Sericulture Center, Tak Province, Thailand. The fresh stems were chopped and dried. Then, the dried plant was extracted by maceration technique using 80% ethanol for 2 cycles at room temperature. The crude extract was filtered through 0.45*µ*m pore size nylon membrane (VertiClean™, Vertical Chromatography Co., Ltd., Bangkok, Thailand). After filtration, the filtrate was evaporated under reduced pressure using a rotary evaporator (BUCHI Rotavapor R – 114, Switzerland) and continued drying using a water bath (M25 LAUDA, Germany). Then, the dried powder was stored in a tight, light-protected container. The dried crude extract was dissolved in Dimethyl Sulfoxide (DMSO) to give a concentration of 100 mg/ml as a stock solution for the determination of antibacterial activity and anti-inflammatory activity of MSE.

### 2.2 Bacteria and Culture Conditions

*P. gingivalis* strain ATCC 33277 and *A. actinomycetemcomitans* strain ATCC 29523 were obtained from the American Type Culture Collection, Manassas, VA, USA. *P. gingivalis* was grown anaerobically at 37°C in an anaerobic chamber under an atmosphere of 80% nitrogen: 10% carbon dioxide: 10% hydrogen. Culture media was ATCC medium 2722 supplemented tryptic soy broth/agar. Tryptic Soy Broth (TBS) Blood Agar Plates (BAP) supplemented per liter were made with the addition of 30.0 g of TBS (Becton, Dickinson and Company, Sparks, MD, USA), 5.0 g of yeast extract (BD), 0.5 g of L-cysteine hydrochloride (HiMedia Laboratories Pvt. Ltd, India), and 15.0 g of agar. Vitamin K1 (Sigma Chemical Co., St. Louis, MO, USA) and hemin (Sigma) were added to give the final concentrations of 1.0 μg/ml and 5 μg/ml, then the medium was autoclaved for 15 min at 121°C. The medium was cooled down to 50°C and horse blood was added in the final concentration of 5% (Oxoid Limited, Cheshire, England). The bacteria were inoculated from BAP into 5 ml of TSB and cultured anaerobically for 18 to 24 h at 37°C, then diluted in the TSB and grown overnight to reach early log phase. *A. actinomycetemcomitans* was grown at 37°C under an atmosphere of 5% carbon dioxide. Culture media was made with 37 g of Brain Heart Infusion (BHI) broth (Becton, Dickinson and Company, Sparks, MD, USA) and 15.0 g of BHI agar. The medium was autoclaved for 15 min at 121°C. Bacteria were inoculated from BHI agar into 5 ml of BHI and cultured for 18 to 24 h at 37°C, 5% CO_2_ then diluted in BHI and grown overnight to reach an early log phase. Bacterial cells were harvested by low-speed centrifugation (4,000 rpm 5 min), washed, and resuspended in 1 ml Phosphate Buffered Saline (PBS), pH 7.2. The concentration of bacteria was determined with a spectrophotometer at an optical density of 625 nm (OD 0.08-0.1 = 1-2x10^8^cfu/ml).

### 2.3 Determination of Antibacterial Activity

The antibacterial activity of MSE was evaluated using a disc diffusion method on an agar medium. *P. gingivalis* was grown an anaerobic TSB blood agar plates in an anaerobic environment at 37°C for 3-5 days. *A. actinomycetemcomitans* was grown in brain heart infusion agar plate to 5% CO_2_ at 37°C for 1-2 days. The MSE stock solution was diluted to 12.5-100 mg/ml at a final concentration and introduced onto the disc and allowed to dry. Then, the disc was impregnated onto the seed agar plate. DMSO was used as a negative control, whereas 0.2% chlorhexidine was used as a positive control. The tests were performed in triplicate. The antibacterial activity was determined based on the diameter of the zone of inhibition.

A broth dilution technique was used to determine the antibacterial activity of MSE (Jorgensen and Turnidge, 2007). The Minimum Inhibitory Concentration (MIC) and the Minimum Bactericidal Concentration (MBC) values of MSE were determined. From MSE stock solution, serial two-fold dilutions (15.625-2,000 μg/mL) were prepared with 1 ml volume with TSB for *P. gingivalis* and 1 ml volume with BHI for *A. actinomycetemcomitans*. Subsequently, each bacterial strain suspension with a final inoculum of 1x106 bacteria/ml was added to the tubes with different dilutions of MSE. The negative control was prepared using 2% DMSO as was maximum concentration was used to dissolve the extracts in antibacterial experiments. Positive control was prepared using 0.2% chlorhexidine. Solvent and medium controls were used for reference. After 24 h incubation, the bacterial growth was observed for turbidity. MIC was defined as the lowest concentration present in a clear well with no turbidity by visual inspection. The MBC assay was performed continuously after MIC assay. An aliquot of 100 μl from dilution tubes without turbidity was inoculated on TSB blood agar plates for *P. gingivalis* and inoculated on BHI agar plate for *A. actinomycetemcomitans*. The plates were then incubated for the appearance of bacterial growth for 3-5 days. The MBC was determined by the lowest concentration of the crude extract that showed no viable growth on the agar plate. The tests were performed in triplicate.

### 2.4 Preparation and Culture of hPDL Fibroblasts

This study was approved by the Naresuan University Institutional Review Board (COA No. 013/2015). hPDL fibroblasts were harvested from caries-free and periodontally healthy teeth extracted for orthodontic reasons with informed consent. After extraction, the teeth were rinsed with PBS several times. After getting rinsed, the periodontal ligament tissues attached to the medial part of the root were removed using a curette. The tissue explants were plated in 35-mm tissue culture dishes and grown in Dulbecco’s Modified Eagle’s Medium (DMEM: Hyclone, South Logan, UT, USA) with 10% Fetal Bovine Serum (FBS) (Hyclone), 2 mM L-glutamine (Hyclone), 100 U/mL penicillin (Hyclone) and 100 µg/mL streptomycin (Hyclone) at 37ºC, with 5% CO_2_, and 95% humidity. After reaching confluence, the hPDL fibroblasts were detached with 0.25% Trypsin-EDTA (Hyclone). The cells between the 3^rd^ and 5^th^ passage were used in the following studies. Three independent experiments with cells from 3 different individuals were performed in each experiment.

### 2.5 Cell Viability Assays

hPDL fibroblasts were seeded into 24-well culture plates at 5 X10^4^ cells per well in DMEM supplemented with 10% FBS for 24h. Then, the medium was replaced with serum-free medium and the cells were treated with various concentrations of MSE or *P. gingivalis* LPS (*Invivo*Gen, San Diego, CA, USA) in serum-free medium for 24 h. The viability of hPDL fibroblasts towards extracts from *M. alba* stem was then measured using the Cell Titer 96 aqueous one solution cell proliferation kit following the manufacturer’s instructions (MTS assay; Promega, Madison, WI, USA). MTS working solution was added to each well and incubated for 5 min at 37ºC. After transferring 200 μl of supernatant to a 96-well plate, the soluble formazan absorbance was recorded using a plate reader at 490 nm. All measurements were performed in triplicate and were repeated on three different occasions (*n* =3). Cytotoxicity was calculated by expressing absorbance values as a percentage of control values (100% represented zero cytotoxicity).

### 2.6. Quantification of Cytokines mRNA by Reverse Transcription Real-Time PCR

hPDL fibroblasts were seeded in a 6-well plate at a density of 5x10^5^ cells per well. Following 1 h of incubation with 1.25, 2.5, 5.0 μg/ml MSE or 0.05% DMSO, the cells were stimulated with 10 μg/ml *P. gingivalis* LPS for 23 h. Total RNA was then isolated by Nucleospin RNAII (Macherey-Nagel GmbH&Co. KG) according to the manufacturer’s instruction. Concentration and purity of RNA were determined using a Nano Drop ND-2000c spectrophotometer (Thermo Fisher Scientific, Wilmington, MA, USA). cDNA was synthesized from 1 µg of total RNA and mixed with LightCycler 480 DNA SYBR Green I Master (Roche). Two-step quantitative –reverse transcription PCR was performed in duplicate using the LightCycler 480 II Real-Time PCR System (Roche). The hot start enzyme was activated (95 °C for 5 min), and cDNA was then amplified for 40 cycles consisting of denaturation at 94 °C for 20 s and annealing/extension at 60 °C for 20 s. A melt curve assay was then performed (65 °C for 1 min and then the temperature was increased by 1.1°C every 10 s) to detect the formation of primer-derived trimers and dimers. Glyceraldehyde-3-phosphate dehydrogenase (GAPDH) was used as a control. The sequences of the PCR primers were used as follows: IL-6, (forward) 5′-CCTGAACCTTCCAAAGATGGC-3′, and (reverse) 5′-CTGACCAGAAGAAGGAATGCC-3′ [13]; IL-8, (forward) 5′-CGATGTCAGTGCATAAAGACA-3′, and (reverse) 5′- TGAATTCTCAGCCCTCTTCAAA-3′ [13]; and GAPDH, (forward) 5′-TGAAGGTCGGAGTCAACGGAT-3′, and (reverse) 5′-TCACACCCATGACGAACATGG-3′ [13]. Data were analyzed with the Light Cycler^®^ 480 software version 1.5. The average starting quantity of fluorescence units was used for the analysis. Quantification was calculated by using GAPDH for internal control.

### 2.7. Measurement of IL-6 and IL-8 Concentrations by ELISA

After hPDL fibroblasts were stimulated with 10 μg/ml *P. gingivalis* LPS and treated with MSE for 24 h, the supernatants were collected and the levels of IL-6 and IL-8 protein were measured using the human IL-6 and IL-8 ELISA kits according to the manufacturer’s protocol (BioLegend, San Diego, CA, USA).The optical density of each well at 450 nm was determined using a microplate reader (BIO-RAD Laboratories, Hercules, CA, USA). All measurements were performed in duplicate and repeated at three different occasions. The mean values of the two measurements were used for statistical analysis.

### 2.8 Statistical Analysis

Statistical analyses were performed using *SPSS* software version 19.0. All data were expressed as the mean ± Standard Deviation (SD). Differences between the experimental groups were analyzed with the analysis of variance (ANOVA) followed by a post-hoc Tukey test in all experiments. The differences were considered to be significant when *p*<0.05.

## 3 RESULTS

### 3.1 Growth Inhibition of Periodontopathic Bacteria by MSE

Two putative periodontal pathogens were selected for the antibacterial analysis. As shown in Table **1**, the disc diffusion analysis revealed that 50 mg/ml MSE showed antibacterial activity against *P. gingivalis* and *A. actinomycetemcomitans* with inhibition zones of 10.00 ± 0.33 mm and 17.33 ± 0.58 mm, respectively. The positive control, 0.2% chlorhexidine, showed an inhibition zone of10.33 ± 0.58 mm and 18.33 ± 0.58 mm against the strains of *P. gingivalis* and *A. actinomycetemcomitans*, respectively. Next, MIC and MBC of MSE against these two periodontal pathogens were employed by broth dilution technique to evaluate their antibacterial strength. As reported in Table **2**, the MIC indicated bacteriostatic concentration against *P. gingivalis* and *A. actinomycetemcomitans* in the average value of 62.5 µg/ml and 250µg/ml, respectively. MSE showed potentially bactericidal activity against *P. gingivalis* with an MBC of 62.5 µg/ml while an MBC of *A. actinomycetemcomitans* reached to 500 µg/ml. On the other hand, MIC and MBC values of 0.2% chlorhexidine against both pathogenic bacteria were found to be effective with a concentration of 1.25 µg/ml and 2.5 µg/ml, respectively. The results obtained from three repetitions for each bacterium were similar.

### 3.2 Cell Viability of hPDL Fibroblasts Treated With MSE

**Fig. (1):**
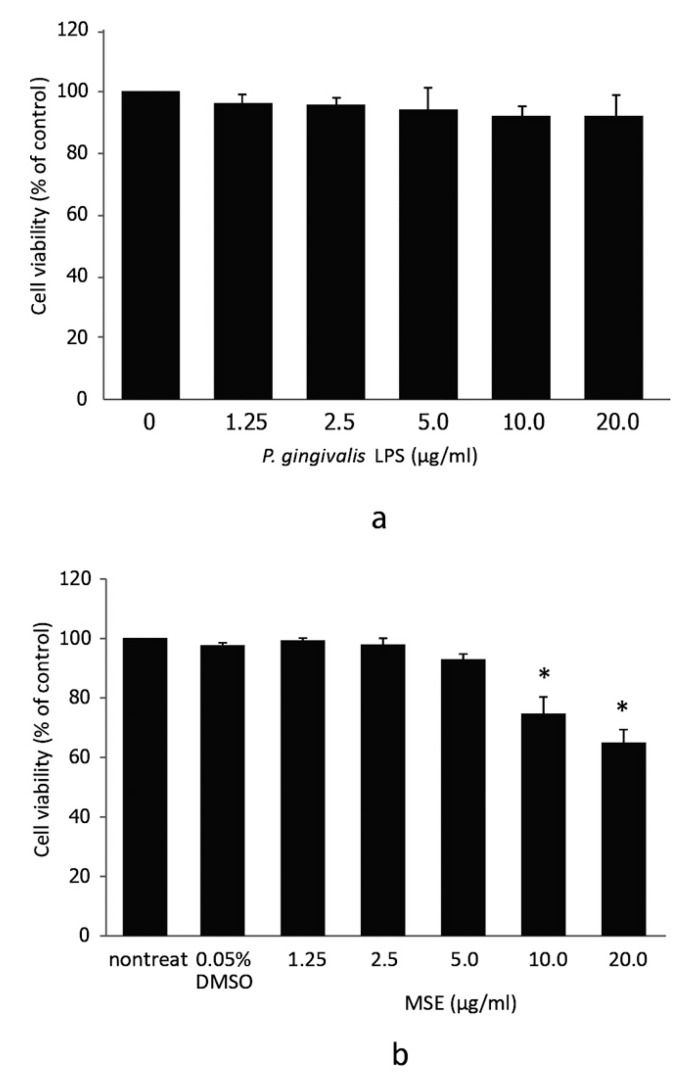
Effect of *P. gingivalis* LPS (a) and MSE (b) on cell viability in hPDL fibroblasts. Cells were treated with various concentrations and cell viability was measured by MTS assay at 24 h after culture. The data are expressed as the percentage of viable cells compared with the nontreated cells. The values (mean ± SD) are the average of three independent experiments performed in triplicate; * indicate significant (*p* < 0.05).

Initially, we investigated the possible cytotoxic effects of *P. gingivalis* LPS and MSE by treating hPDL fibroblasts with different concentrations of *P. gingivalis* LPS or MSE (0, 1.25, 2.5, 5.0, 10.0 and 20.0 µg/ml) for 24 h followed by MTS assay. Dose-dependent adverse effects of *P. gingivalis* LPS and MSE on the viability of hPDL fibroblast were detected. *P. gingivalis* LPS tested here showed no effect on hPDL fibroblasts viability (Fig. **1a**). MSE at a concentration between 0 and 5.0 µg/ml was not toxic to hPDL fibroblasts as the cell viability was not significantly altered compared to the non-treated control. However, at the concentration of 10 to 20 µg/ml, cell viability significantly decreased indicating that cells died at these concentrations (Fig. **1b**).Thus, MSE was used at less than 5.0 μg/ml for following experiments.

### 3.3 Inhibition of the LPS-Induced Expressions of IL-6 and IL-8 mRNA by MSE

**Fig. (2):**
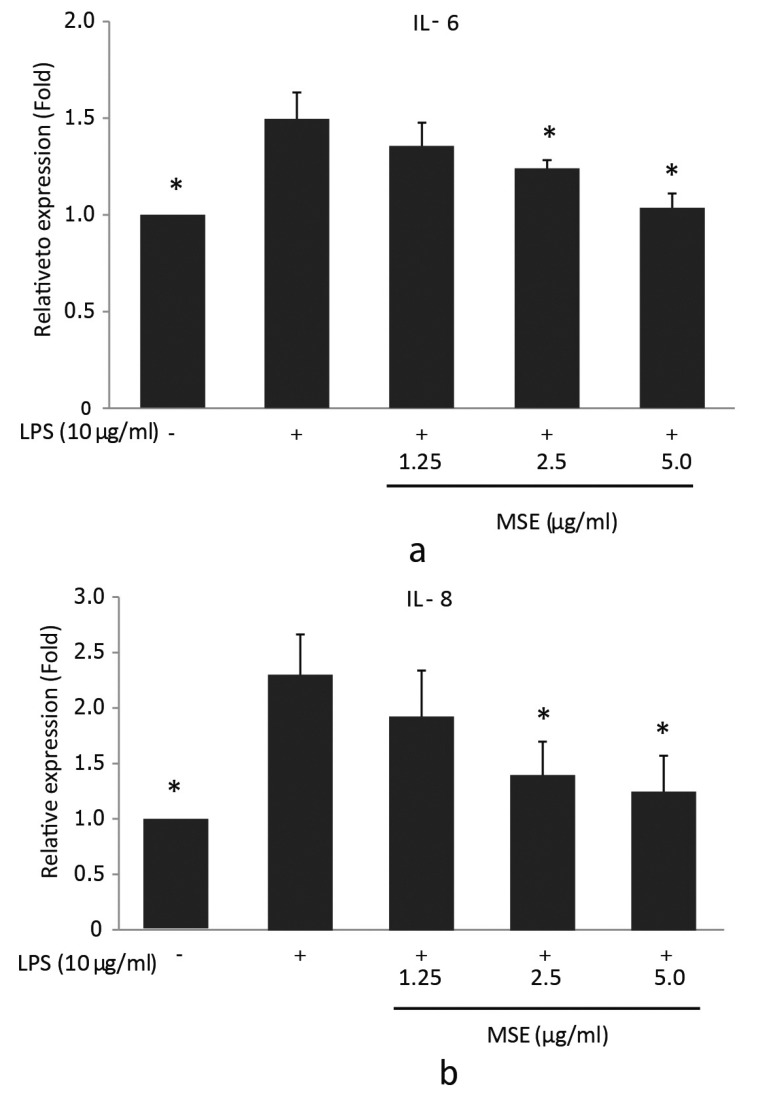
MSE suppressed IL-6 (a) and IL-8 (b) mRNA expressionin hPDL fibroblasts. Cells were incubated with 1.25, 2.5 and 5.0 µg/ml MSE or 0.05% DMSO for 1 h, followed by 10 μg/ml *P. gingivalis* LPS stimulation. After 24 h, cells were extracted for Real-time PCR. Levels of IL-6 and IL-8 mRNA were normalized to GAPDH. The values (mean ± SD) are the average of three independent experiments performed in triplicate; *indicate significant difference from *P. gingivalis* LPS treatment only (*p* < 0.05).

In order to investigate the inhibitory effect of MSE on *P. gingivalis* LPS-induced IL-6 and IL-8 mRNA expression, reverse transcription q-PCR was performed. hPDL fibroblasts were treated with MSE for 1 hour prior to stimulation with *P. gingivalis*-LPS for 23 h. The results showed that MSE inhibited gene expression of IL-6 and IL-8 in a dose dependent manner compared to fibroblasts untreated with MSE. The concentrations 2.5 and 5.0 µg/ ml significantly (*p* < 0.05) inhibited the expression of both IL-6 (Fig. **2a**) and IL-8 (Fig. **2b**) mRNA compared to cells treated with *P. gingivalis* LPS alone. These results suggest that the inhibitory effect of MSE on *P. gingivalis* LPS-induced IL-6 and IL-8 mRNA expression was regulated at the transcription.

### 3.4 MSE Inhibits LPS Induced IL-6 and IL-8 Production

**Fig. (3):**
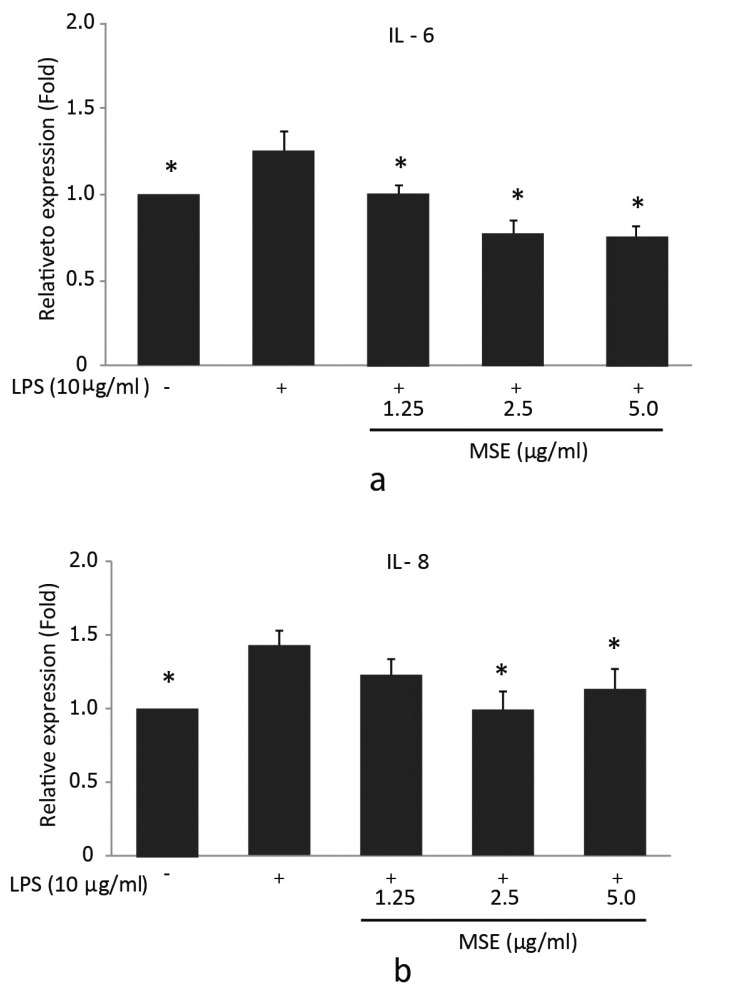
MSE suppressed IL-6 (a) and IL-8 (b) secretion in hPDL fibroblasts. Cells were incubated with 1.25, 2.5 and 5.0 µg/ml MSE or 0.05% DMSO for 1 h, followed by 10 μg/ml *P. gingivalis* LPS stimulation. The culture supernatants were collected 24 h later. The amount of IL-6 and IL-8 protein expression were measured by ELISA. The values (mean ± SD) are the average of three independent experiments performed in triplicate; * indicate significant difference from *P. gingivalis* LPS treatment only (*p* < 0.05).

To further confirm the inhibitory effect of the MSE on IL-6 and IL-8 secretion by *P. gingivalis* LPS-stimulated hPDL fibroblasts, ELISA was used to detect the levels of these cytokines in supernatants. For IL-6, the significantly (*p* < 0.05) lower cytokine concentrations compared to the cells treated with *P. gingivalis* LPS were detected after incubation with 1.25, 2.5 and 5.0 µg/ml MSE (Fig. **3a**). However, IL-8 levels showed a significant decrease in the cells incubated with 2.5 and 5.0 µg/ml MSE compared to the cells treated with *P. gingivalis* LPS alone (Fig. **3b**). MSE suppressed *P. gingivalis* LPS induced IL-6 and IL-8 production in a concentration-dependent manner. These findings indicated that MSE could also inhibit the expression of pro-inflammatory cytokines IL-6 and IL-8 at the protein levels.

## 4 DISCUSSION

The present study shows for the first time that the addition of MSE inhibited the growth of periodontal pathogens and reduced the expression of pro-inflammatory cytokines by hPDL fibroblasts stimulated by *P. gingivalis* LPS. *M. alba* stem extract is one of the important natural sources of oxyresveratrol, which has been reported to possess numerous pharmacological properties, especially its anti-inflammatory [15], hypoglycemic [18], antioxidant [18, 19], and anti-microbial activities [19].

Our results showed that MSE possesses antibacterial activities against two major bacterial causes of periodontitis, *P. gingivalis* and *A. actinomycetemcomitans*. However, *P. gingivalis* showed more sensitivity to MSE than *A. actinomycetemcomitans*. According to the literature reports, we presume that the main active substance which contributed to the antibacterial and anti-inflammation activities could be oxyresveratrol. A previous report has indicated that oxyresveratrol from *Artocarpuslakoocha* [13] and *M. alba* leaf extract exhibited antibacterial activities against *P. gingivalis* as well as *A. actinomycetemcomitans* [20]. *P. gingivalis* was reported to be the most sensitive organism against *M. alba* leaf extract with an MIC value of 1.95 mg/ml, while *A. actinomycetemcomitans* was less sensitive with an MIC value of 3.91 mg/ml [18]. Our results are in accordance with the previous studies reported the inhibitory effect of *M. alba* extract on these two Gram-negative bacteria with variation in MIC and MBC values. A variation in the sensitivities of these plant extracts may be due to variation in the method of extraction, bioactive compounds as well as bacterial strains used, and variation in chemical constituents. However, the present study only evaluated antibacterial activity against the selected pathogens in planktonic but not in a biofilm state. A complex biofilm composed of multiple species is generally more resistant to antimicrobial agents than planktonic cells [21]. The diversity and metabolic state of microorganisms in a subgingival biofilm play key roles in antibiotic resistance and the inability of the bacterial community to be phagocytized by host inflammatory cells, eventually leads to periodontal disease. Further studies on the potential of MSE to control the growth of periodontal pathogens in biofilm state are necessary to clarify its effects.

IL-6 and IL-8 are important cytokines involved in the immune responses against infection, but overexpression of these cytokines might cause inflammatory diseases, including periodontal diseases [22]. Previous studies have reported that *P. gingivalis* LPS stimulated the production of pro-inflammatory cytokines secreted from human gingival fibroblasts [23, 24]. In the present study, it was also found that *P. gingivalis* LPS stimulation increased the expression of IL-6 and IL-8 from hPDL fibroblasts in both mRNA and protein levels. The results showed that MSE extracts inhibited the secretion of IL-6 and IL-8 from *P. gingivalis* LPS-induced hPDL fibroblasts in a dose-dependent manner. A recent study has reported that MSE demonstrated anti-inflammatory activity through the inhibition of nitric oxide production in LPS-stimulated RAW 264.7 cells [15]. Consistent with the previous studies, the reduced levels of *P. gingivalis* LPS-induced expression of inflammatory cytokines in the present study indicated that MSE tested here may have potent anti-inflammatory effects. The effective concentration of MSE for anti-inflammatory activity is 2.5 µg/ml. This concentration did not affect the hPDL fibroblasts viability at 24 h, suggesting the primary effect is the suppression of cytokine production rather than cytotoxicity. However, the MIC of the extract against *P. gingivalis* and *A. actinomycetemcomitans* was 62.5 µg/mL and 250 µg/mL, respectively. These concentrations induced cytotoxic effects in hPDL fibroblasts in 24 h. Therefore, further investigations of the anti-inflammatory effects of MSE and signaling pathways related to inflammation in hPDL fibroblasts or other oral cells as well as time-kill assays on the periodontopathic bacteria are needed to define the optimal concentration for clinical application.

## CONCLUSION

The present results show that MSE has an anti-bacterial potential against the two putative periodontal pathogens. In addition, MSE can inhibit *P. gingivalis* LPS-induced production of the pro-inflammatory cytokine, IL-6 and IL-8 expression by hPDL fibroblasts. These findings indicate that MSE may be used as an adjunctive agent for periodontal treatment, but these observations require further experimental verification of the effects.

## Figures and Tables

**Table 1:** Inhibition zone of MSE against periodontal pathogens.

Periodontal Pathogens	Zone of Inhibition (mm)	
	50 mg/ml MSE	0.2% Chlorhexidine
*P. gingivalis*	10.00 ± 0.33	10.33 ± 0.58
*A. actinomycetemcomitans*	17.33 ± 0.58	18.33 ± 0.58

Data are means of three replicates (n = 3) ± standard error.

**Table 2:** MICs and MBCs of MSE against the periodontal pathogens.

Periodontal Pathogens	MIC (µg/ml)		MBC (µg/ml)	
	MSE	0.2%Chlorhexidine	MSE	0.2%Chlorhexidine
*P. gingivalis*	62.5	1.25	62.5	2.5
*A. actinomycetemcomitans*	250	1.25	500	2.5
